# Effect of Lifestyle Counselling via a Mobile Application on Disease Activity Control in Inflammatory Arthritis: A Single-Blinded, Randomized Controlled Study

**DOI:** 10.3390/nu16101488

**Published:** 2024-05-14

**Authors:** Türker Kurt, Diana Vossen, Falk Schumacher, Johannes Strunk, Dmytro Fedkov, Christine Peine, Felix Lang, Abdullah Khalil, Ralph Brinks, Stefan Vordenbäumen

**Affiliations:** 1 Department of Rheumatology, St. Elisabeth-Hospital Meerbusch-Lank, Hauptstr. 74-76, 40668 Meerbusch, Germany; 2 Department of Rheumatology and Hiller Research Unit, University Hospital Düsseldorf, Medical Faculty, Heinrich Heine University Düsseldorf, Moorenstr. 5, 40225 Düsseldorf, Germany; 3 Rheumazentrum Ruhrgebiet, Ruhr University Bochum, Claudiusstrasse 45, 44649 Herne, Germany; 4 Department of Rheumatology, Krankenhaus Porz am Rhein, 51149 Cologne, Germany; 5 Faculty of Health, School of Medicine, Witten/Herdecke University, 58455 Witten, Germany; 6 Midaia GmbH, 69123 Heidelberg, Germany; dmytro.fedkov@midaia.com (D.F.);; 7 Department of Internal Medicine 3, Bogomolets National Medical University, 01601 Kyiv, Ukraine; 8 Medical Biometry and Epidemiology, University of Witten/Herdecke, 58448 Witten, Germany

**Keywords:** Mediterranean Diet, healthy lifestyle, rheumatoid arthritis, axial spondyloarthritis, psoriatic arthritis

## Abstract

Background: Mobile applications (apps) are a resource for information on lifestyle and nutrition which are associated to improved outcomes in inflammatory arthritis. Objective: The aim of this study was to explore whether targeted lifestyle counselling via an app improves disease activity in arthritis patients. Methods: Patients with rheumatoid arthritis (RA), spondyloarthritis (SpA), psoriatic arthritis (PsA) were randomized to 12 weeks of lifestyle counselling via an app (Mida, Midaia GmbH, Germany) pertaining to a healthy Mediterranean Diet, physical activity, and mental health. Disease activity was measured with specific instruments by a blinded physician and categorized (remission, low, moderate, high). Dietary adherence was assessed by the Mediterranean Diet Adherence Screener (MEDAS). Mixed effects logistic regression adjusted to baseline disease activity, age, and sex were calculated. Results: Of 158 patients included (73% female, 53.3 ± 11.7 years), 74 were in the active counselling group (ACG). All showed improvement in low disease activity or remission. ACG patients had an odds ratio (OR) of 2.8 (95%-CI 1.1–7.2, *p* = 0.035), while OR in the control group was not significant OR = 2.1 (0.9–5.0, *p* = 0.097). The control group was less likely to reach a MEDAS >= 4 (OR = 0.16 (0.03–0.77), *p* = 0.02), while this was not seen in the ACG (OR = 0.54 (0.06–4.63), *p* = 0.6). Patients in the ACG showed a tendency towards improved adhesion to a Mediterranean Diet (MEDAS) (β = 0.35 (−0.05–0.74), *p* = 0.086). This tendency was not observed in the control group (β = 0.09 (−0.29–0.46), *p* = 0.64). Conclusions: Individualized lifestyle and dietary counselling via app may help to improve disease control in inflammatory arthritis patients.

## 1. Introduction

Rheumatoid arthritis (RA), psoriatic arthritis (PsA), and axial spondyloarthritis (axSpA) are the most common autoimmune inflammatory rheumatic diseases which primarily affect joints [1,2,3,4]. They are associated with a considerable socioeconomic burden, including disability, inability to work, and socioeconomic decline [1,5,6,7]. These conditions are considered to develop in individuals with a vulnerable genetic background in conjunction with environmental factors [8,9,10]. In particular, nutritional patterns, physical exercise, and mental health were reported to impact inflammatory joint diseases [11,12,13]. Amongst potentially healthy dietary patterns, a Mediterranean Diet has most consistently been associated with a favorable disease course [14,15,16,17]. Consequently, the European League against Rheumatism (EULAR) recently summarized the evidence and issued recommendations concerning lifestyle modifications in patients with inflammatory rheumatological conditions [12]. Lifestyle changes, however, are notoriously hard to induce in patients [18]. There are several potential obstacles to efficient lifestyle counselling, including time restrictions and insufficient reimbursement modalities or even a lack thereof, depending on the local health policy [19,20,21]. Mobile phone applications (apps) are increasingly used to obtain health information or to obtain patient reported outcomes (PROMs) by arthritis patients and physicians, respectively [22,23]. Even though daily smart phone use is very common amongst patients, regular use of apps intended to improve arthritis is rather scarce [24]. We were therefore interested in exploring if individualized lifestyle counselling by an app could improve disease activity control in arthritis patients, and in identifying the potentially most versatile lifestyle components in this context. 

## 2. Methods

### 2.1. Patients and Study Design

Consecutive rheumatology outpatients with inflammatory arthritis, i.e., RA, PsA, or axSpA from St. Elisabeth-Hospital Meerbusch-Lank, and Krankenhaus Porz am Rhein, Cologne, Germany were recruited to the single-blinded, randomized controlled study from June 2022 until June 2023. Inclusion criteria consisted of eligibility to use and possession of a smart phone, at least 18 years of age, and confirmed diagnosis of an arthritis condition according to either 2010 EULAR criteria for RA [25], 2006 Classification criteria for Psoriatic Arthritis (CASPAR) criteria for PsA [26], or 2011 Assessment of SpondyloArthritis international Society (ASAS) ASDAS criteria for axSpA [27]. Epidemiological data, medication, and disease activity were measured via disease-specific instruments and then categorized into remission, low, moderate, or high disease activity at inclusion and week 12 (Rheumatoid Arthritis Disease Activity Index (RADAI) for RA [28], Disease Activity in PSoriatic Arthritis (DAPSA) score for PsA [29], Bath Ankylosing Spondylitis Disease Activity Index (BASDAI) for axSpA [30]).

Patients were alternatingly randomized to either report PROMs only (control group), or to additionally receive 12 weeks of individualized lifestyle counselling via an app (Mida Rheuma^®^ app version 1 (Midaia GmbH, Heidelberg, Germany), detailed below) pertaining to a healthy Mediterranean Diet, sports and physical activity, mental health, and non-smoking (active counselling group). Patient allocation was blinded to the treating physician. The study applied to the Declaration of Helsinki. All patients gave full informed consent. The study was approved by the Ethics Committee of the Medical Faculty of Heinrich-Heine-University Düsseldorf, Germany (study number 2021-1408). The study was retrospectively registered in the ISRCTN registry (#ISRCTN17343801).

### 2.2. Objectives and Endpoints

The primary study objective was to evaluate the effect of 12-week lifestyle counselling via app on patients’ disease activity by evaluating the number of patients who achieved low disease activity or remission measured using RADAI, DAPSA, or BASDAI. Secondary endpoints were to evaluate the effect of 12-week lifestyle counselling via app on: (1) adherence to Mediterranean Diet measured using Mediterranean Diet Adherence Screener (MEDAS) [31], (2) health-related quality of life measured via Short Form 36 Health Survey (SF36) [32]; (3) physical activity level measured via the Physical Activity, Exercise, and Sport Questionnaire (‘Bewegungs- und Sportaktivität Fragebogen’ (BSA)) [33], and depression and anxiety levels measured via the Patient Health Questionnaire 4 (PHQ 4) [34,35].

### 2.3. Mobile Phone Application

Mida Rheuma App (Midaia, Germany) was used to assess PROM in both groups, or to additionally offer lifestyle counselling in the active counselling group. The Mida Rheuma App is part of the Midaia Software, version 1, which is CE-certified software (UDI-DI 4260731370018) to monitor disease burden and treat patients with RA, PsA, or axSpA using a personalized approach to improve disease activity and physical impairment. The conformity with general safety and performance requirements based on clinical data has been demonstrated previously [22]. The evaluation of the clinical performance of the Midaia Software was based on the general safety and performance requirements and verification according to the Medical Devise Regulation. The software comprises (1) the Conversational Agent which collects patient data on lifestyle factors, mental health, and medication using standardized questionnaires. Based on this, personal behaviors that influence disease activity and physical impairment are identified. They are cross-checked with recommendations from medical guidelines [11,12,13,27,36,37,38,39,40,41,42,43] and medical standards for app development as issued by EULAR [44] to match patients with a personalized therapy plan (series of treatment action plans). The Midaia algorithm uses this data as a basis for determining the order of prescribing interventions. (2) Treatment Action Plans consist of consecutive and prioritized lifestyle suggestions and provide motivation to achieve behavioral changes compatible with recommendations. The application of a specific action plan is determined by a supervised learning algorithm that manages the personalized assignment of action plans. This algorithm employs a multifaceted baseline assessment, using key indicators such as age, sex, diagnosis, dietary and mental health profiles, level of social interaction, patient’s understanding of disease and treatment modalities, functional status of the musculoskeletal system, physical activity levels, disease activity, fatigue, harmful habits, sleep quality, and others. These indicators are analyzed to establish a prioritized, initial array of tailored action plans, each uniquely suited to the individual patient’s profile. This process integrates machine learning techniques, including categorical and quantitative variable analysis, to split and prune the initial set of plans. Subsequently, a dynamic weighting system is employed, wherein each plan is assigned coefficients based on two crucial criteria: the urgency of the patient’s needs (e.g., the disparity between the patient’s current nutritional habits and the recommended anti-inflammatory diet) and the potential impact of the intervention (according to the levels of evidence). Notably, certain plans receive higher priority coefficients, especially those targeting mental health improvements, as these have been shown to enhance overall plan efficacy and patient adherence [45,46,47]. The action plans are offered and explained to the patients in a chat mode.

The algorithm is further enhanced by a continuous feedback loop, where the effectiveness of individual and combined plans is constantly monitored and analyzed. This feedback informs real-time adjustments to the coefficients and hierarchical ranking of the plans. Additionally, the system employs patient clustering algorithms to identify specific demographic and personal characteristic clusters that respond more favorably to certain intervention strategies.

**Figure 1:**
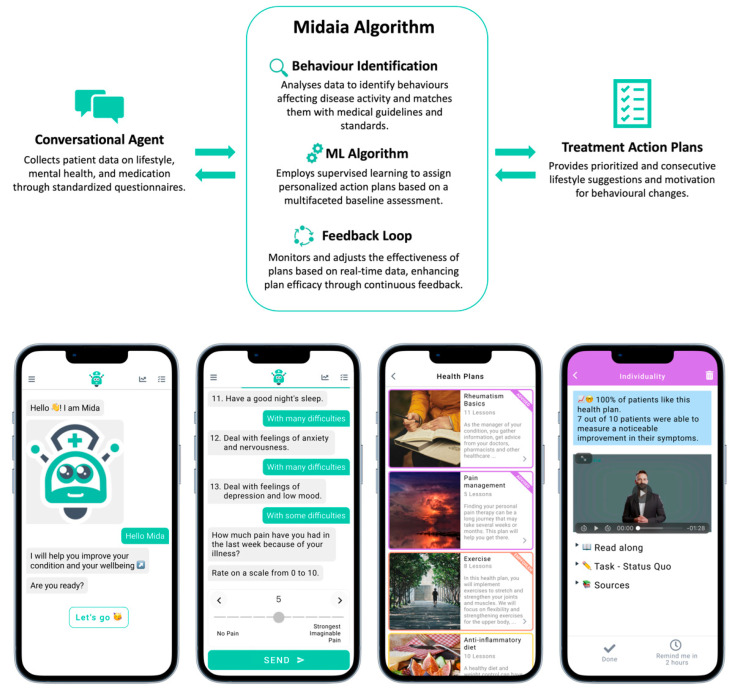
Graphical summary of the counselling strategy via app (**upper part**), and representative screenshots of the app during lifestyle counselling (**lower part**).

Recommendations can include diet, exercise, mental health, social life, coping with the disease, pain and fatigue management, joint protection, motivation, self-efficacy and self-management, smoking cessation, and medication adherence. Each area of care contains several action plans (a total of 44 action plans are available), for example, a mental group consisting of cognitive behavioral therapy, mindfulness meditation, body scan meditation, support in depression management, gratefulness, and improved sleep. Action plans are 7 to 11 days long, give evidence-based tips, help, and motivation, and break the recommendations into sub-tasks to achieve the behavior change. Each action plan contains detailed information on general terminology, an up-to-date, evidence-based view of the topic, and specific step-by-step call to actions, which are presented in texts, diagrams, pictures, and video courses. For example, in the exercise section, depending on the patient’s needs and the degree of disease activity and functional status, exercises to overcome morning stiffness, specific training sessions (different for patients with axial and peripheral arthritis) of three degrees of complexity, or a yoga course developed with the participation of an experienced yoga instructor and a rheumatologist may be recommended. Patients are reminded to fulfill the recommended tasks daily, and the implemented recommendation’s effect is monitored and controlled. The counselling strategy of the app along with representative screenshots from counselling sessions is illustrated in Figure 1.

### 2.4. Statistical Analysis

R version 4.3.2 was used for statistical analysis. Demographic and baseline characteristics were summarized using standard descriptive statistics, including sample size. For continuous variables, mean and SD are provided in cases where skewness and kurtosis were between −2 and 2; otherwise, median and range are provided; for categorical variables, numbers and percentages are provided.

The primary outcome parameter was low disease activity or remission at week 12. Analysis was by logistic mixed linear modelling with patient ID as the random effect, thereby also accounting for bias due to regression to the mean (e.g., especially in cases of highly active patients). An additional exploratory analysis was similarly performed for a MEDAS > 4. Results are reported as odds ratios (ORs) with 95% confidence intervals (CI). Furthermore, exploratory analyses were also performed via mixed linear modelling with patient identity as the random effect for health-related quality of life domains of SF36, weight, self-estimated time doing sports per week, exercise per week according to BSA, MEDAS in all patients, PHQ, and DAS28 in a subgroup of RA patients as outcome variables. Results are reported as the regression coefficient (β) with 95% confidence intervals.

All models were calculated adjusted for age and sex. As sensitivity analyses, models were also calculated without any adjustments and with an additional adjustment for education as a proxy for social status. Educational levels were categorized from lowest (1) to highest (5) based on the highest diploma within the German schooling system (Middle School (‘Hauptschule’), High School (‘Realschule’), High School with higher qualification (‘Fachhochschulreife’), High School with university qualification (‘Abitur’), or university degree). In case dissimilarities were observed between the models, these findings are reported in the Section 3. Complete case analysis was performed. There was no prior sample size estimation due to the exploratory nature of the study. Groups were analyzed according to whether or not lifestyle counselling was provided via the app (active counselling group) or not (inactive control group). A *p*-value ≤ 0.05 was considered statistically significant.

## 3. Results

### 3.1. Patients’ Characteristics

**Figure 2:**
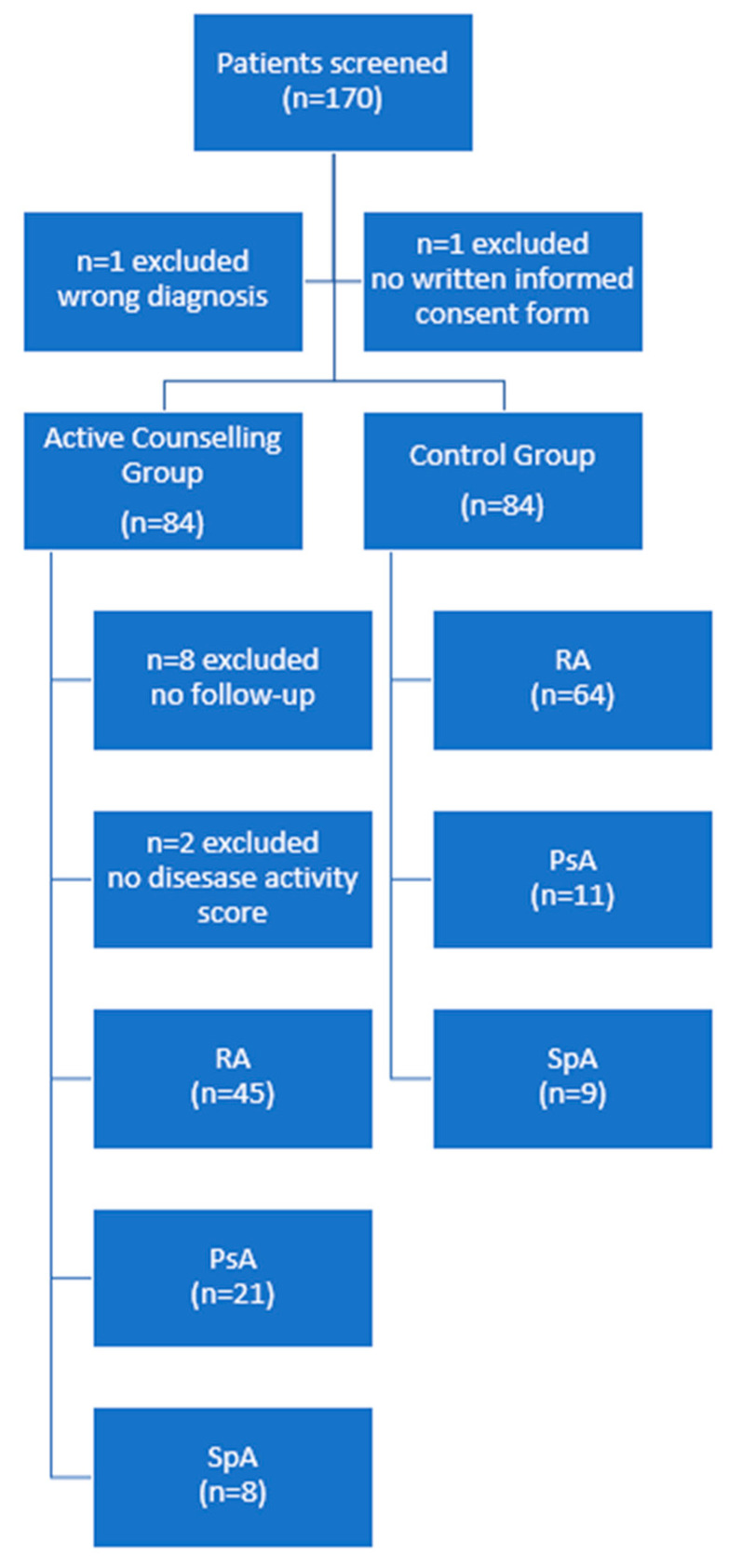
Patient flow chart. RA, rheumatoid arthritis; PsA, psoriatic arthritis; SpA, axial spondyloarthritis.

A total of 170 patients were screened for inclusion and 168 were alternatingly randomized to the active counselling or control group (PROM only). After exclusion of ineligible cases, 158 patients were included in the study, of whom 74 were allocated to the active counselling group, and 84 to the control group (Figure 2). Patients’ characteristics are summarized in Table 1. For a subgroup of 43 RA patients, DAS28 measurements were available for additional analyses (17 in the active counselling group (age 56.2 ± 8.4 years, 73% female, disease duration 9.2 ± 8.6 years), 26 in the control group (age 54.7 ± 11.0 years, 72.6% female, disease duration 13.2 ± 9.6 years)). Treatment regimens were variable across groups, most commonly methotrexate (41.8%), other conventional synthetic disease modifying antirheumatic drugs (16.5%), TNF inhibitors (34.8%), Janus kinase inhibitors (9.5%), Interleukin (IL) 6 inhibitors (7.6%), and IL-17 inhibitors (5.1%). Under 5% of total patients received abatacept, IL23 inhibitors, or rituximab. There were no obvious disparities between the control group and the active counselling group.

### 3.2. Disease Activity Assessment

**Figure 3:**
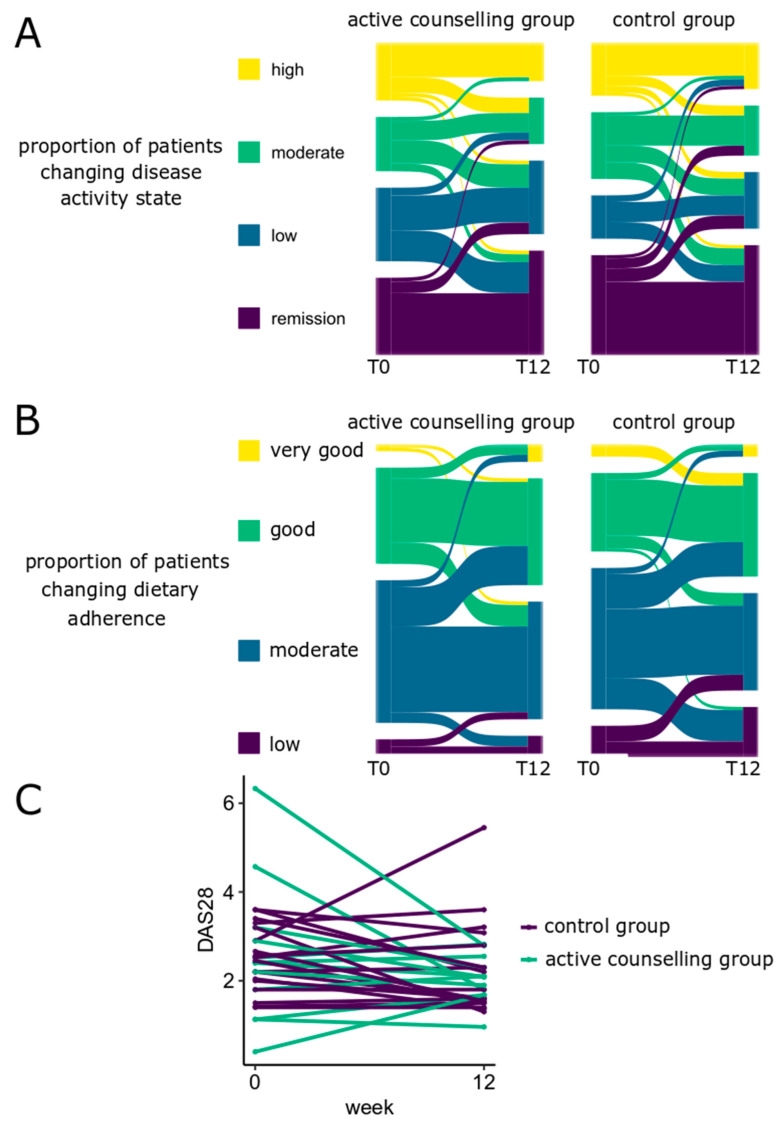
Outcome parameters at baseline and after 12 weeks of active counselling via app. (**A**,**B**) The diagrams show the proportion of patients who changed to a different disease state (**A**) or dietary adherence state (**B**) from the start (T0) to the end of the study after 12 weeks (T12). The thickness of the line is proportional to the number of patients changing the respective state. For instance, in the active counseling group, the proportion of patients downgrading the disease activity state is greater than in the control group (**A**). Disease activity is categorized based on disease-specific instruments (**A**), and dietary adherence (**B**) to a healthy diet measured via the Mediterranean Diet Adherence Screener (MEDAS) and categorized into low (0–3), moderate (4–6), good (7–9), or very good (10–12) adherence of 158 patients with inflammatory arthritis. (**C**). Disease activity was measured via the Disease Activity Score 28 (DAS28) in the subgroup of 43 patients with rheumatoid arthritis at the start and end of the study after 12 weeks.

In order to assess the effect of lifestyle counselling via the app on disease activity in inflammatory arthritis, the odds ratio (OR) of achieving low disease activity or remission were calculated in the active counselling and the control group. We observed a significantly increased OR with a substantial effect size to enter low disease activity or remission (OR = 2.8 (95%-CI 1.1–7.2), *p* = 0.035). In the control group, this trend was not significant (OR = 2.1 (0.9–5.0), *p* = 0.097). The results are depicted in Figure 3A.

In a subgroup of RA patients, serial DAS28 measurements were available. Similarly, RA patients in the active counselling group showed a borderline significant decline in DAS28 (β = −0.6 (−1.3 to 0), *p* = 0.055), while this trend was not significant in the control group (β = −0.4 (−0.9 to 0.14), *p* = 0.17). In the fully adjusted model including adjustments for social status, there was a significant decline in the active counselling group (β = −0.6 (−1.3 to −0.04), *p* = 0.046), while the control group showed no significant change (β = −0.4 (−0.9 to 0.08), *p* = 0.13). The results are depicted in Figure 3C.

### 3.3. Change in Lifestyle Parameters Including Adherence to Mediterranean Diet

Next, we were interested in exploring which lifestyle parameters were mainly associated with positive effects on disease activity states.

Concerning adherence to a healthy Mediterranean Diet, we compared the MEDAS values (Figure 3B). Patients in the active counselling group showed a tendency towards improved MEDAS scores (β = 0.35 (−0.05–0.74), *p* = 0.086). This tendency was not observed in the control group (β = 0.09 (−0.29–0.46), *p* = 0.64). Furthermore, we previously reported that a MEDAS ≥ 4 may represent a reasonable cut-off to discern healthy from a rather unhealthy diet in the German population of patients with inflammatory rheumatic diseases [48]. Indeed, in the present study, the control group was less likely to reach a MEDAS ≥ 4 (OR = 0.16 (0.03–0.77), *p* = 0.02), while this tendency was not seen in the active counselling group (OR = 0.54 (0.06–4.63), *p* = 0.6). This association was similarly observed in the fully adjusted model also incorporating a proxy for social status, albeit to a lesser extent (control group: OR = 0.28 (0.28–1.2), *p* = 0.087; active counselling group: OR = 0.84 (0.12–5.82), *p* = 0.86). No change was observed with the weight development in either group (active counselling: β = −0.1 (−0.69–0.49), *p* = 0.75, control group: β = 0.08 (−0.47–0.63), *p* = 0.78).

Concerning sports and physical activity, no differences were observed in the weekly time spent for physical activity (active counselling group: β = 0.03 (−0.28–0.34), *p* = 0.85; control group: β = −0.09 (−0.38–0.2), *p* = 0.54), or the BSA exercise questionnaire (active counselling group: β −13.1 (−61.2–34.8), *p* = 0.59; control group: β = −6.3 (−51.6–39.3), *p* = 0.79).

Concerning psychic wellbeing, no differences were observed in the depression screening scale PHQ4 (active counselling group: β = 0.23 (−0.25–0.71), *p* = 0.34; control group: β = 0.23 (−0.22–0.69), *p* = 0.32)

### 3.4. Changes in Health-Related Quality of Life

Finally, we explored whether changes in health-related quality of life parameters as measured by the SF36 were observable in any of the groups. In the course of the 12-week observational period, there were no significant changes in the following parameters in either the active counselling or the control group: Physical Function (active counselling: β = 0.7 (−2.4–3.9), *p* = 0.6; control: β = −0.2 (−3.2–2.7), *p* = 0.9), Physical Role Functioning (active counselling: β = 3.0 (−5.5–11.7), *p* = 0.5; control: β = 2.3 (−5.8–10.4), *p* = 0.6), Emotional Role Functioning (active counselling: β = 0.8 (−8.6–10.3), *p* = 0.9; control: β = 3.9 (−5.0–12.8), *p* = 0.4), Vitality (active counselling: β = 0.6 (−2.9–4.0), *p* = 0.8; control: β = −0.2 (−3.5–3.0), *p* = 0.9), Mental Health (active counselling: β = −1.0 (−4.3–2.3), *p* = 0.5; control: β = 1.6 (−1.5–4.7), *p* = 0.3), Social Role Functioning (active counselling: β = −1.4 (−6.2–3.5), *p* = 0.6; control: β = 1.6 (−2.9–6.1), *p* = 0.5), Bodily Pain (active counselling: β = 3.4 (−1.2–8.0), *p* = 0.2; control: β = −0.6 (−4.9–3.8), *p* = 0.8), or General Health (active counselling: β = −2.2 (−5.6–1.2), *p* = 0.2; control: β = −1.5 (−4.7–1.7), *p* = 0.4). The same was true for the summarizing parameters Physical Summary Component (active counselling: β = 0.7 (−3.0–4.4), *p* = 0.7; control: β = 0.5 (−3.0–3.9), *p* = 0.8); and the Mental Summary Component (active counselling: β = −0.4 (−4.4–3.7), *p* = 0.9; control: β = 1.8 (−2.0–5.6), *p* = 0.3).

## 4. Discussion

In the present pilot study, patients with inflammatory rheumatoid joint diseases were randomized to receive individualized lifestyle counselling pertaining to a healthy Mediterranean Diet (in particular, action plans aimed at the implementation of a Mediterranean Diet, weight loss, explaining the pro-inflammatory and anti-inflammatory properties of certain foods, forming the consumption of less meat, and the consumption of more vegetables, fruits, whole grains, and vegetable oils with a high content of omega-3 polyunsaturated fatty acids), physical activity, and mental health via a mobile phone application or to the retrieval of PROMs only. Patients in the active counselling group had significantly increased odds of reaching at least low disease activity at a considerable effect size. The study was performed in the most common arthritic joint diseases as a whole and not limited to a specific disease. However, a similar trend was noted in the largest group of patients with RA only, employing a disease-specific parameter as the outcome criterion. Moreover, the effects observed were robust for adjustments with potential confounders such as age, sex, and education as a proxy for the social status, or the activity status at disease onset.

The lifestyle counselling via the app used in this study administered advice on various domains, including diet, physical activity, and mental health. This is in line with the recently issued EULAR recommendations on lifestyle behavior in rheumatic diseases which stress that lifestyle choices should ideally be assessed as a whole rather than separately [11]. In an attempt to explore which of the lifestyle domains targeted might have the largest versatility, we identified adherence to a healthy diet. In this context, we considered a Mediterranean Diet to represent a healthy nutritional choice, as this diet is chiefly advocated to carry health benefits [49]. Patients may also benefit from other dietary patterns such as a traditional Asian diet [49], gluten-free and vegan diet, or fasting [50]. We did not account for these alternative potentially healthy dietary patterns, because we considered the latter pattern to likely be the most common amongst the healthy dietary patterns in Germany [51,52], and due to practicality issues. Moreover, based on a previous study, we defined a MEDAS of 4 or more to represent a sufficiently healthy diet [48]. Of note, the adherence to a healthy Mediterranean Diet is generally more pronounced in southern European countries, with a mean MEDAS up to 8 in Spain, but as low as 4 to 5 in Bulgaria, North America, South America, or Australia [53,54,55]. The German population generally follows a Western rather than a Mediterranean Diet [56] with median MEDAS as low as 3 points [51]. In follow-up studies, the inclusion of a more detailed dietary assessment is likely to provide further insight and help to even better define healthy nutrients for rheumatic diseases beyond broad categories such as that of a ‘healthy Mediterranean Diet’.

We hypothesize, however, that the specific method of counselling via an app may considerably influence the effectiveness, as some studies with different apps found no health benefits concerning, e.g., improvements in diet or physical activity [57], or improvements in quality of life measures [58] which were not obvious in our study. In this context, individualized counselling and goal setting were previously identified as factors promoting a successful health app [59]. Previous studies on apps have shown that mental health issues [60] and physical activity status [61,62] may also be improved by apps. In the present pilot study, we did not use instruments fit to sensitively detect more subtle changes in both domains. Hence, we cannot exclude that clinically significant effects on mental health and physical activity went unnoticed in the active counselling group due to the method of detection, or due to the small sample size.

An unsolved issue when advising patients to use apps for health benefits is the potential to promote smart phone addiction [63]. We did not address this issue in our current study. Reassuringly, no such increased risk for dependency was found in a previous study on the topic [62]. A further limitation consists in the exploratory approach of the study with a lack of reliable data for sample size estimations.

## 5. Conclusions

In summary, we found evidence of improved disease control and adherence to a healthy diet by individualized counselling via an app in arthritis patients. Further randomized controlled trials on these topics are warranted.

## Figures and Tables

**Table 1:** Patients’ characteristics.

Parameter	All	Active Counselling	Control Group
	n (%) or Mean ± SD	n (%) or Mean ± SD	n (%) or Mean ± SD
	or Median (Range)	or Median (Range)	or Median (Range)
Female	115 (72.8%)	54 (43%)	61 (72.6%)
Age	53.3 ± 11.7	53.4 ± 12.0	53.2 ± 11.5
Disease duration	8 (1–40)	8 (1–34)	8 (1–40)
Education level			
1	29 (18.4%)	12 (16.2%)	17 (20.2%)
2	44 (27.8%)	20 (27%)	24 (28.6%)
3	30 (19%)	17 (23%)	13 (15.5.%)
4	18 (11.4%)	7 (9.5%)	11 (13.1%)
5	37 (23.4%)	18 (24.3%)	19 (22.6%)
Disease			
RA	109 (69%)	45 (60.8%)	64 (76.2%)
PsA	32 (20.3%)	21 (28.4%)	11 (13.1%)
axSpA	17 (10.7%)	8 (10.8%)	9 (10.7%)
Activity State			
Remission	53 (33.5%)	22 (29.7%)	31 (36.9%)
Low	33 (20.9%)	19 (25.7%)	14 (16.7%)
Moderate	39 (24.7%)	16 (21.6%)	23 (27.4%)
High	33 (20.9%)	17 (23%)	16 (19%)
Weight (kg)	82.6 ± 19.2	81.2 ± 16.8	83.9 ± 21.1
HRQoL			
PF	62 ± 27.1	61.4 ± 28.4	62.5 ± 26.1
RP	39.1 ± 41.3	47.3 ± 41.9	31.8 ± 39.6
RE	55.1 ± 44.3	57.7 ± 43.6	52.8 ± 45.2
VT	42.4 ± 20.6	43.1 ± 20.7	41.8 ± 20.5
MH	63.7 ± 18.9	65.8 ± 18.8	62.7 ± 19.0
SF	67.5 ± 25.7	69.4 ± 25.3	65.8 ± 26.0
BP	52.7 ± 25.4	53.7 ± 26.3	51.8 ± 24.7
GH	45.7 ± 17.4	47.8 ± 17.5	43.9 ± 17.1
PCS	49.9 ± 23.2	52.5 ± 24.5	47.5 ± 21.8
MCS	57.2 ± 23.0	59.0 ± 23.1	47.5 ± 21.8
BSA	0 (0–1800)	0 (0–750)	0 (0)
Sport/week (h)	2 (1–8)	2 (1–8)	2 (1–8)
MEDAS	6 ± 2	6.2 ± 1.9	5.8 ± 2.1
PHQ4	3.2 ± 2.6	3.0 ± 2.4	3.4 ± 2.8
CRP	2.2 (0–133)	2.35 (0–133)	2.1 (0–61.9)
DAS28*	2.55 (0.4–7.5)	2.52 (0.4–7.5)	2.55 (0.96–19.2)

Age and disease duration in years. Educational levels were categorized from lowest (1) to highest (5) based on the highest within the German schooling system (see methods for details). RA, rheumatoid arthritis; PsA, psoriatic arthritis; axSpA, axial spondyloarthriits; Activity State categorized according to disease specific instrument (see methods); HRQoL, health-related quality of life according to short form 36 (PF, physical functioning; RP, role-functioning physical; RE role-function emotional; VT, vitality; MH, mental health; SF, social functioning; BP, body pain; GH, general health; PCS, physical component summary; MCS, mental component summary); BSA, Physical Activity, Exercise, and Sport Questionnaire (‘Bewegungs- und Sportaktivität Fragebogen’); MEDAS, Mediterranean Diet Adherence Screener; PHQ4, depression and anxiety levels measured by Patient Health Questionnaire 4; CRP, c-reactive protein; DAS28*, Disease Activity Score with 28 joints in patients with rheumatoid arthritis only.

## Data Availability

The data presented in this study are available upon reasonable request from the corresponding author due to potential intellectual property issues.

## Abbreviations

RA	Rheumatoid Arthritis
PsA	Psoriatic Arthritis
axSpA	Axial Spondyloarthritis
EULAR	European League against Rheumatism
app	Mobile phone application
PROM	Patient Reported Outcomes
CASPAR	2006 Classification criteria for Psoriatic ARthritis
RADAI	Rheumatoid Arthritis Disease Activity Index
DAPSA	Disease Activity in PSoriatic Arthritis (DAPSA)
BASDAI	Bath Ankylosing Spondylitis Disease Activity Index (BASDAI)
MEDAS	Mediterranean Diet Adherence Screener
SF36	Short Form 36 Health Survey for Health-Related Quality of Life
BSA	Physical Activity, Exercise, and Sport Questionnaire (‘Bewegungs- und Sportaktivität Fragebogen’ (BSA))
PHQ4	Patient Health Questionnaire 4 for depression and anxiety level assessment
SD	Standard Deviation
OR	Odds Ratios
CI	Confidence Intervals

## References

[1] Michelsen B., Fiane R., Diamantopoulos A.P., Soldal D.M., Hansen I.J.W., Sokka T., Kavanaugh A., Haugeberg G. (2015). A Comparison of Disease Burden in Rheumatoid Arthritis, Psoriatic Arthritis and Axial Spondyloarthritis. PLoS ONE.

[2] Di Matteo A., Bathon J.M., Emery P. (2023). Rheumatoid Arthritis. Lancet.

[3] Ritchlin C.T., Colbert R.A., Gladman D.D. (2017). Psoriatic Arthritis. N. Engl. J. Med..

[4] Sieper J., Poddubnyy D. (2017). Axial Spondyloarthritis. Lancet.

[5] Ciofoaia E.I., Pillarisetty A., Constantinescu F. (2022). Health Disparities in Rheumatoid Arthritis. Ther. Adv. Musculoskelet..

[6] Boehncke W.-H., Menter A. (2013). Burden of Disease: Psoriasis and Psoriatic Arthritis. Am. J. Clin. Dermatol..

[7] Malinowski K.P., Kawalec P. (2015). The Indirect Costs of Ankylosing Spondylitis: A Systematic Review and Meta-Analysis. Expert Rev. Pharmacoecon Outcomes Res..

[8] Deane K.D., Demoruelle M.K., Kelmenson L.B., Kuhn K.A., Norris J.M., Holers V.M. (2017). Genetic and Environmental Risk Factors for Rheumatoid Arthritis. Best Pract. Res. Clin. Rheumatol..

[9] Talotta R., Atzeni F., Sarzi-Puttini P., Masala I.F. (2019). Psoriatic Arthritis: From Pathogenesis to Pharmacologic Management. Pharmacol. Res..

[10] Liao H.-T., Tsai C.-Y., Lai C.-C., Hsieh S.-C., Sun Y.-S., Li K.-J., Shen C.-Y., Wu C.-H., Lu C.-H., Kuo Y.-M. (2022). The Potential Role of Genetics, Environmental Factors, and Gut Dysbiosis in the Aberrant Non-Coding RNA Expression to Mediate Inflammation and Osteoclastogenic/Osteogenic Differentiation in Ankylosing Spondylitis. Front. Cell Dev. Biol..

[11] Gwinnutt J.M., Wieczorek M., Cavalli G., Balanescu A., Bischoff-Ferrari H.A., Boonen A., de Souza S., de Thurah A., Dorner T.E., Moe R.H. (2022). Effects of Physical Exercise and Body Weight on Disease-Specific Outcomes of People with Rheumatic and Musculoskeletal Diseases (RMDs): Systematic Reviews and Meta-Analyses Informing the 2021 EULAR Recommendations for Lifestyle Improvements in People with RMDs. RMD Open.

[12] Gwinnutt J.M., Wieczorek M., Balanescu A., Bischoff-Ferrari H.A., Boonen A., Cavalli G., de Souza S., de Thurah A., Dorner T.E., Moe R.H. (2022). 2021 EULAR Recommendations Regarding Lifestyle Behaviours and Work Participation to Prevent Progression of Rheumatic and Musculoskeletal Diseases. Ann. Rheum. Dis..

[13] Wieczorek M., Gwinnutt J.M., Ransay-Colle M., Balanescu A., Bischoff-Ferrari H., Boonen A., Cavalli G., de Souza S., de Thurah A., Dorner T.E. (2022). Smoking, Alcohol Consumption and Disease-Specific Outcomes in Rheumatic and Musculoskeletal Diseases (RMDs): Systematic Reviews Informing the 2021 EULAR Recommendations for Lifestyle Improvements in People with RMDs. RMD Open.

[14] Shekhar K.V., Pathak M.M., Pisulkar G. (2023). Diet and Lifestyle Impact on Rheumatoid Arthritis: A Comprehensive Review. Cureus.

[15] Schäfer C., Keyßer G. (2022). Lifestyle Factors and Their Influence on Rheumatoid Arthritis: A Narrative Review. J. Clin. Med..

[16] Caso F., Navarini L., Carubbi F., Picchianti-Diamanti A., Chimenti M.S., Tasso M., Currado D., Ruscitti P., Ciccozzi M., Annarumma A. (2020). Mediterranean Diet and Psoriatic Arthritis Activity: A Multicenter Cross-Sectional Study. Rheumatol. Int..

[17] Ortolan A., Felicetti M., Lorenzin M., Cozzi G., Ometto F., Striani G., Favero M., Doria A., Ramonda R. (2023). The Impact of Diet on Disease Activity in Spondyloarthritis: A Systematic Literature Review. Jt. Bone Spine.

[18] Ogdie A., Asch D.A. (2020). Changing Health Behaviours in Rheumatology: An Introduction to Behavioural Economics. Nat. Rev. Rheumatol..

[19] Kiestra L., de Vries I.A.C., Mulder B.C. (2020). Determinants of Lifestyle Counseling and Current Practices: A Cross-Sectional Study among Dutch General Practitioners. PLoS ONE.

[20] Ampt A.J., Amoroso C., Harris M.F., McKenzie S.H., Rose V.K., Taggart J.R. (2009). Attitudes, Norms and Controls Influencing Lifestyle Risk Factor Management in General Practice. BMC Fam. Pract..

[21] Geense W.W., van de Glind I.M., Visscher T.L.S., van Achterberg T. (2013). Barriers, Facilitators and Attitudes Influencing Health Promotion Activities in General Practice: An Explorative Pilot Study. BMC Fam. Pract..

[22] Fedkov D., Berghofen A., Weiss C., Peine C., Lang F., Knitza J., Kuhn S., Krämer B.K., Leipe J. (2022). Efficacy and Safety of a Mobile App Intervention in Patients with Inflammatory Arthritis: A Prospective Pilot Study. Rheumatol. Int..

[23] Richter J.G., Nannen C., Chehab G., Acar H., Becker A., Willers R., Huscher D., Schneider M. (2021). Mobile App-Based Documentation of Patient-Reported Outcomes—3-Months Results from a Proof-of-Concept Study on Modern Rheumatology Patient Management. Arthritis Res. Ther..

[24] MacBrayne A., Curzon P., Soyel H., Marsh W., Fenton N., Pitzalis C., Humby F. (2023). Attitudes towards Technology Supported Rheumatoid Arthritis Care: Investigating Patient- and Clinician-Perceived Opportunities and Barriers. Rheumatol. Adv. Pract..

[25] Aletaha D., Neogi T., Silman A.J., Funovits J., Felson D.T., Bingham C.O., Birnbaum N.S., Burmester G.R., Bykerk V.P., Cohen M.D. (2010). 2010 Rheumatoid Arthritis Classification Criteria: An American College of Rheumatology/European League Against Rheumatism Collaborative Initiative. Ann. Rheum. Dis..

[26] Taylor W., Gladman D., Helliwell P., Marchesoni A., Mease P., Mielants H. (2006). Classification Criteria for Psoriatic Arthritis: Development of New Criteria from a Large International Study. Arthritis Rheum..

[27] Rudwaleit M., van der Heijde D., Landewe R., Akkoc N., Brandt J., Chou C.T., Dougados M., Huang F., Gu J., Kirazli Y. (2011). The Assessment of SpondyloArthritis International Society Classification Criteria for Peripheral Spondyloarthritis and for Spondyloarthritis in General. Ann. Rheum. Dis..

[28] Fransen J., Langenegger T., Michel B.A., Stucki G. (2000). Feasibility and Validity of the RADAI, a Self-Administered Rheumatoid Arthritis Disease Activity Index. Rheumatology.

[29] Schneeberger E.E., Citera G., Nash P., Smolen J.S., Mease P.J., Soriano E.R., Helling C., Szumski A.E., Mundayat R., de León D.P. (2023). Comparison of Disease Activity Index for Psoriatic Arthritis (DAPSA) and Minimal Disease Activity (MDA) Targets for Patients with Psoriatic Arthritis: A Post Hoc Analysis of Data from Phase 3 Tofacitinib Studies. Semin. Arthritis Rheum..

[30] van der Heijde D., Deodhar A., Fleischmann R., Mease P.J., Rudwaleit M., Nurminen T., Davies O. (2017). Early Disease Activity or Clinical Response as Predictors of Long-Term Outcomes with Certolizumab Pegol in Axial Spondyloarthritis or Psoriatic Arthritis. Arthritis Care Res..

[31] Hebestreit K., Yahiaoui-Doktor M., Engel C., Vetter W., Siniatchkin M., Erickson N., Halle M., Kiechle M., Bischoff S.C. (2017). Validation of the German Version of the Mediterranean Diet Adherence Screener (MEDAS) Questionnaire. BMC Cancer.

[32] Ware J., Kosinski M., Keller S.D. (1996). A 12-Item Short-Form Health Survey: Construction of Scales and Preliminary Tests of Reliability and Validity. Med. Care.

[33] Fuchs R., Klaperski S., Gerber M., Seelig H. (2015). Messung der Bewegungs- und Sportaktivität mit dem BSA-Fragebogen: Eine methodische Zwischenbilanz. Z. Gesundheitspsychol..

[34] Kroenke K., Spitzer R.L., Williams J.B.W., Monahan P.O., Löwe B. (2007). Anxiety Disorders in Primary Care: Prevalence, Impairment, Comorbidity, and Detection. Ann. Intern. Med..

[35] Löwe B., Wahl I., Rose M., Spitzer C., Glaesmer H., Wingenfeld K., Schneider A., Brähler E. (2010). A 4-Item Measure of Depression and Anxiety: Validation and Standardization of the Patient Health Questionnaire-4 (PHQ-4) in the General Population. J. Affect Disord..

[36] Combe B., Landewe R., Daien C.I., Hua C., Aletaha D., Álvaro-Gracia J.M., Bakkers M., Brodin N., Burmester G.R., Codreanu C. (2017). 2016 Update of the EULAR Recommendations for the Management of Early Arthritis. Ann. Rheum. Dis..

[37] Rausch Osthoff A.-K., Niedermann K., Braun J., Adams J., Brodin N., Dagfinrud H., Duruoz T., Esbensen B.A., Günther K.-P., Hurkmans E. (2018). 2018 EULAR Recommendations for Physical Activity in People with Inflammatory Arthritis and Osteoarthritis. Ann. Rheum. Dis..

[38] Rudwaleit M., van der Heijde D., Landewe R., Listing J., Akkoc N., Brandt J., Braun J., Chou C.T., Collantes-Estevez E., Dougados M. (2009). The Development of Assessment of SpondyloArthritis International Society Classification Criteria for Axial Spondyloarthritis (Part II): Validation and Final Selection. Ann. Rheum. Dis..

[39] Smolen J.S., Landewé R.B.M., Bijlsma J.W.J., Burmester G.R., Dougados M., Kerschbaumer A., McInnes I.B., Sepriano A., van Vollenhoven R.F., de Wit M. (2020). EULAR Recommendations for the Management of Rheumatoid Arthritis with Synthetic and Biological Disease-Modifying Antirheumatic Drugs: 2019 Update. Ann. Rheum. Dis..

[40] van der Heijde D., Ramiro S., Landewé R., Baraliakos X., Van den Bosch F., Sepriano A., Regel A., Ciurea A., Dagfinrud H., Dougados M. (2017). 2016 Update of the ASAS-EULAR Management Recommendations for Axial Spondyloarthritis. Ann. Rheum. Dis..

[41] Zangi H.A., Ndosi M., Adams J., Andersen L., Bode C., Boström C., van Eijk-Hustings Y., Gossec L., Korandová J., Mendes G. (2015). EULAR Recommendations for Patient Education for People with Inflammatory Arthritis. Ann. Rheum. Dis..

[42] Nikiphorou E., Santos E.J.F., Marques A., Böhm P., Bijlsma J.W., Daien C.I., Esbensen B.A., Ferreira R.J.O., Fragoulis G.E., Holmes P. (2021). 2021 EULAR Recommendations for the Implementation of Self-Management Strategies in Patients with Inflammatory Arthritis. Ann. Rheum. Dis..

[43] Deutsche Gesellschaft für Ernährung 10 Rules of the Germand Society for Nutrition. https://www.dge.de/gesunde-ernaehrung/gut-essen-und-trinken/dge-empfehlungen/.

[44] Najm A., Nikiphorou E., Kostine M., Richez C., Pauling J.D., Finckh A., Ritschl V., Prior Y., Balážová P., Stones S. (2019). EULAR Points to Consider for the Development, Evaluation and Implementation of Mobile Health Applications Aiding Self-Management in People Living with Rheumatic and Musculoskeletal Diseases. RMD Open.

[45] Jun E.R., Kim S.H., Cho Y.J., Kim Y.-A., Lee J.Y. (2019). The Influence of Negative Mental Health on the Health Behavior and the Mortality Risk: Analysis of Korean Longitudinal Study of Aging from 2006 to 2014. Korean J. Fam. Med..

[46] Rebar A.L., Taylor A. (2017). Physical Activity and Mental Health; It Is More than Just a Prescription. Ment. Health Phys. Act..

[47] Lo Moro G., Corezzi M., Bert F., Buda A., Gualano M.R., Siliquini R. (2023). Mental Health and Adherence to Mediterranean Diet among University Students: An Italian Cross-Sectional Study. J. Am. Coll. Health.

[48] Vordenbäumen S., Kleefisch M., Sokolowski A., Düsing C., Richter J.G., Brinks R., Schneider M., Chehab G. (2023). Beneficial Effects Associated to a Healthy Lifestyle in Systemic Lupus Erythematosus: A Cross-Sectional Study. Lupus.

[49] Cena H., Calder P.C. (2020). Defining a Healthy Diet: Evidence for the Role of Contemporary Dietary Patterns in Health and Disease. Nutrients.

[50] Alunno A., Carubbi F., Bartoloni E., Grassi D., Ferri C., Gerli R. (2021). Diet in Rheumatoid Arthritis versus Systemic Lupus Erythematosus: Any Differences?. Nutrients.

[51] Neumann F.A., Jagemann B., Makarova N., Börschel C.S., Aarabi G., Gutmann F., Schnabel R.B., Zyriax B.-C. (2022). Mediterranean Diet and Atrial Fibrillation: Lessons Learned from the AFHRI Case–Control Study. Nutrients.

[52] Galbete C., Kröger J., Jannasch F., Iqbal K., Schwingshackl L., Schwedhelm C., Weikert C., Boeing H., Schulze M.B. (2018). Nordic Diet, Mediterranean Diet, and the Risk of Chronic Diseases: The EPIC-Potsdam Study. BMC Med..

[53] García-Conesa M.-T., Philippou E., Pafilas C., Massaro M., Quarta S., Andrade V., Jorge R., Chervenkov M., Ivanova T., Dimitrova D. (2020). Exploring the Validity of the 14-Item Mediterranean Diet Adherence Screener (MEDAS): A Cross-National Study in Seven European Countries around the Mediterranean Region. Nutrients.

[54] Hutchins-Wiese H.L., Bales C.W., Porter Starr K.N. (2022). Mediterranean Diet Scoring Systems: Understanding the Evolution and Applications for Mediterranean and Non-Mediterranean Countries. Br. J. Nutr..

[55] Quarta S., Massaro M., Chervenkov M., Ivanova T., Dimitrova D., Jorge R., Andrade V., Philippou E., Zisimou C., Maksimova V. (2021). Persistent Moderate-to-Weak Mediterranean Diet Adherence and Low Scoring for Plant-Based Foods across Several Southern European Countries: Are We Overlooking the Mediterranean Diet Recommendations?. Nutrients.

[56] Leonhäuser I.-U., Dorandt S., Willmund E., Honsel J. (2004). The Benefit of the Mediterranean Diet. Eur. J. Nutr..

[57] Stuber J.M., Mackenbach J.D., de Bruijn G.-J., Gillebaart M., Hoenink J.C., Middel C.N.H., de Ridder D.T.D., van der Schouw Y.T., Smit E.G., Velema E. (2024). Real-World Nudging, Pricing, and Mobile Physical Activity Coaching Was Insufficient to Improve Lifestyle Behaviours and Cardiometabolic Health: The Supreme Nudge Parallel Cluster-Randomised Controlled Supermarket Trial. BMC Med..

[58] Dobies B., White A.J., Isberg A., Gudmundsson S.F., Oddsson S. (2024). Digital Health Program Improves Quality of Life in Rheumatoid Arthritis: A Retrospective Analysis of Real-World Data. Clin. Exp. Rheumatol..

[59] Manskow U.S., Sagelv E.H., Antypas K., Zanaboni P. (2023). Adoption, Acceptability and Sustained Use of Digital Interventions to Promote Physical Activity among Inactive Adults: A Mixed-Method Study. Front. Public Health.

[60] Peuters C., Maenhout L., Cardon G., De Paepe A., DeSmet A., Lauwerier E., Leta K., Crombez G. (2024). A Mobile Healthy Lifestyle Intervention to Promote Mental Health in Adolescence: A Mixed-Methods Evaluation. BMC Public Health.

[61] Cai J., Li G. (2023). Exercise or Lie down? The Impact of Fitness App Use on Users’ Wellbeing. Front. Public Health.

[62] Liu J. (2024). Promoting a Healthy Lifestyle: Exploring the Role of Social Media and Fitness Applications in the Context of Social Media Addiction Risk. Health Educ. Res..

[63] Jeong G.-C. (2016). Relationships among Mental Health, Internet Addiction, and Smartphone Addiction in University Students. J. Korea Contents Assoc..

